# Subacute-Onset Anemia Following COVID-19 Vaccine Combination with ChAdOx (AstraZeneca) and BNT162b2 (BioNTech, Pfizer)—A Case Report

**DOI:** 10.3390/reports9030254

**Published:** 2026-08-04

**Authors:** Konstantina Salveridou, Theodoros Tzamalis, Sabine Haase, Aristoteles Giagounidis

**Affiliations:** 1 Department of Oncology, Hematology and Palliative Care, Marien Hospital, 40479 Düsseldorf, Germany; 2 Department of Oncology, Hematology and Palliative Care, Rheinland Klinikum Neuss GmbH, 41464 Neuss, Germany

**Keywords:** COVID-19, vaccination, erythropoiesis, pure red cell aplasia, anemia, erythropoietin, bone marrow

## Abstract

**Background and Clinical Significance**: The COVID-19 pandemic led to the rapid development of effective vaccination strategies. Although COVID-19 vaccines are generally safe, rare hematological adverse events have been reported, most prominently vaccine-induced immune thrombotic thrombocytopenia (VITT). Isolated cases of autoimmune cytopenias and bone marrow failure syndromes following COVID-19 vaccination have also been described. **Case Presentation:** We report the case of an 80-year-old male who developed subacute-onset severe normocytic anemia with reticulocytopenia and mild leukopenia following heterologous COVID-19 vaccination with ChAdOx1 nCoV-19 (AstraZeneca) and BNT162b2 (Pfizer–BioNTech). Seven days after the second vaccination, mild anemia was detected, progressing over the following weeks to symptomatic anemia requiring hospitalization. Extensive diagnostic evaluation revealed no evidence of hemolysis, nutritional deficiency, autoimmune disease, or viral infection, including SARS-CoV-2 and Parvovirus B19. Bone marrow examination demonstrated an erythroid maturation arrest at the proerythroblast stage, resembling a pure red cell aplasia (PRCA)-like pattern. Cytogenetic and molecular analyses excluded myelodysplastic syndromes. Treatment with erythropoietin resulted in complete hematologic recovery. **Conclusions:** This case suggests that, in rare instances, COVID-19 vaccination may be temporally associated with transient suppression of erythropoiesis. Further studies are required to elucidate underlying mechanisms and to guide diagnosis and management.

## 1. Introduction and Clinical Significance

The COVID-19 pandemic profoundly affected global health, prompting the rapid development and widespread implementation of vaccination programs. COVID-19 vaccines have demonstrated excellent safety profiles; however, rare hematological adverse events have been reported. The most prominent is vaccine-induced immune thrombotic thrombocytopenia (VITT), primarily associated with adenoviral vector vaccines [1]. Sporadic cases of immune-mediated cytopenias, including autoimmune hemolytic anemia (AIHA), immune thrombocytopenia (ITP), and aplastic anemia, have also been described following vaccination [2,3]. In addition, immune thrombocytopenia has been reported after adenoviral vector vaccination, including ChAdOx1-based regimens [4].

Pure red cell aplasia (PRCA) is a rare disorder characterized by normocytic normochromic anemia, severe reticulocytopenia, and selective absence or maturation arrest of erythroid precursors in the bone marrow [5,6]. PRCA is most commonly associated with viral infections—especially Parvovirus B19—but may also occur in autoimmune conditions, in thymoma, or as a drug-induced phenomenon [5,6].

While SARS-CoV-2 infection itself has been shown to affect erythropoiesis and red blood cell morphology [7,8,9,10,11,12,13,14], evidence linking COVID-19 vaccination to PRCA-like bone marrow suppression remains extremely limited. Here, we report a rare case of subacute-onset anemia with erythroid maturation arrest following heterologous COVID-19 vaccination, highlighting diagnostic challenges and potential pathogenetic considerations.

## 2. Case Presentation

An 80-year-old male was admitted on 1 July 2021 due to progressive weakness, dyspnea, and dizziness. His medical history was notable only for hypothyroidism, which was treated with stable levothyroxine therapy; thyroid-stimulating hormone levels were within the reference range. There was no history of autoimmune disease, hematologic disorder, malignancy, toxic exposure, or chronic infection.

The patient received heterologous COVID-19 vaccination consisting of ChAdOx1 nCoV-19 (AstraZeneca) on 17 April 2021, followed by BNT162b2 (Pfizer–BioNTech) on 9 June 2021. Seven days after the second vaccination, mild anemia was detected during routine laboratory testing. Over the following weeks, symptoms progressed, prompting hospital admission (Table 1).

Physical examination was unremarkable. There were no signs of bleeding, lymphadenopathy, hepatosplenomegaly, fever, or B-symptoms. Vital signs were stable.

Laboratory evaluation revealed severe normocytic normochromic anemia with hemoglobin 7.7 g/dL (77 g/L), MCV 87 fL, and marked reticulocytopenia, with a reduced absolute reticulocyte count and reticulocyte production index < 1. Mild leukopenia was present (WBC 2.8 × 10^3^/µL), while platelet count was normal. The patient received two units of packed red blood cells (Table 2).

Hemolysis parameters—including lactate dehydrogenase, bilirubin, and haptoglobin—were within normal limits, and the direct antiglobulin test was negative. Iron studies showed elevated ferritin (490 ng/mL) with increased transferrin saturation (63%), consistent with reduced erythropoiesis rather than iron deficiency. Vitamin B12 and folate levels were normal. Serum erythropoietin was markedly elevated (253 U/L).

Extensive infectious work-up revealed no evidence of viral infection. Parvovirus B19 testing was negative by both serology and PCR. Serologic testing for HIV, hepatitis B and C, Epstein–Barr virus, cytomegalovirus, adenovirus, and influenza A/B was negative. A nasopharyngeal swab was negative for SARS-CoV-2 antigen.

Upper and lower gastrointestinal endoscopy excluded occult bleeding. Peripheral blood smear confirmed anemia with reticulocytopenia and otherwise normal leukocyte morphology.

**Figure 1:**
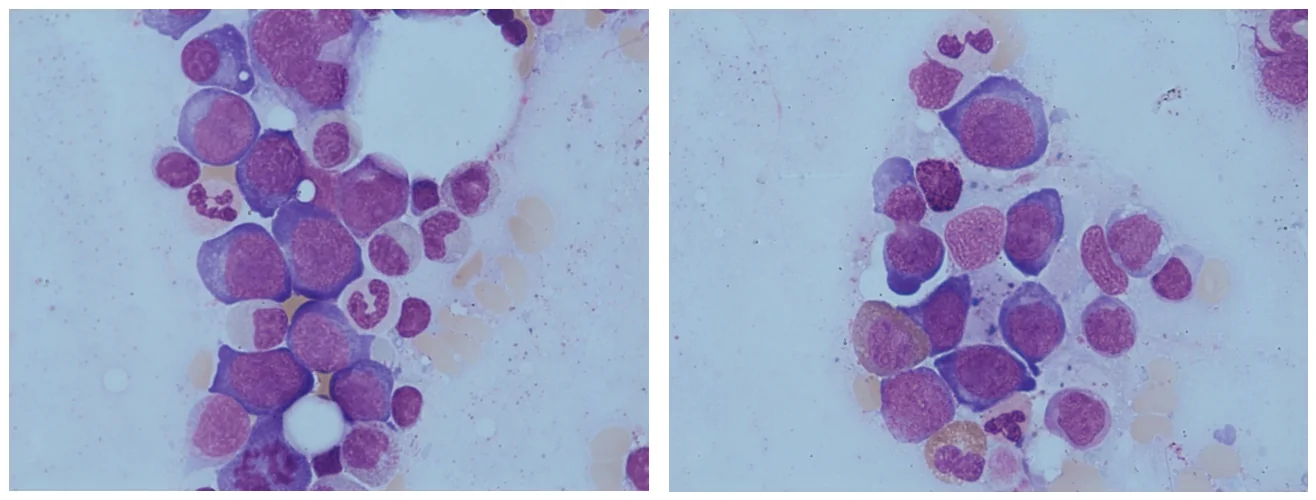
Bone marrow examination.

Bone marrow aspiration demonstrated selective erythroid hypoplasia with maturation arrest at the proerythroblast stage; some proerythroblasts showed cytoplasmic vacuolization, reminiscent of Parvovirus B19-associated changes (Figure 1).

Granulopoiesis and megakaryopoiesis were preserved, and no dysplastic features were observed. Flow cytometry, conventional cytogenetics (46, XY), and next-generation sequencing for myeloid-associated genes revealed no abnormalities (Table 3).

Weekly erythropoietin therapy was initiated, resulting in rapid hematologic recovery. At follow-up on 16 September 2021, hemoglobin had normalized to 15.5 g/dL, and leukocyte counts had fully recovered. Erythropoietin therapy was discontinued after six weeks, with sustained remission.

**Table 1:** Timeline of clinical course.

Date	Event	Findings/Interventions
17 April 2021	First COVID-19 vaccine (ChAdOx1 nCoV-19, AstraZeneca)	No adverse effects
9 June 2021	Second vaccine (BNT162b2, Pfizer–BioNTech)	Tolerated well
16 June 2021	Day 7 post-second dose	Mild anemia detected (routine check-up)
Late June 2021	Progressive weakness/dyspnea	Outpatient evaluation
1 July 2021	Hospital admission	Hb 7.7 g/dL; WBC 2.8 × 10^3^/µL; reticulocytes 4%; platelets normal
July 2021	Diagnostic work-up	BM: erythroid maturation arrest; viral testing negative
July–September 2021	Weekly erythropoietin therapy	Gradual hematologic improvement
16 September 2021	Follow-up	Hb 15.5 g/dL; WBC normalized; therapy discontinued

**Table 2:** Key laboratory findings at admission.

Parameter	Result	Reference Range
**Hemoglobin**	7.7 g/dL	13.5–17.5 g/dL
**Hematocrit**	23%	40–50%
**MCV**	87 fL	80–96 fL
**MCH**	30 pg	27–33 pg
**MCHC**	34 g/dL	32–36 g/dL
**Reticulocytes**	4%	5–20%
**WBC**	2.8 × 10^3^/µL	4.0–10.0 × 10^3^/µL
**Platelets**	186 × 10^3^/µL	150–400
**Ferritin**	490 ng/mL	30–400 ng/mL
**Transferrin**	200 mg/dL	200–360 mg/dL
**Transferrin saturation**	63%	20–50%
**Vitamin B12**	280 pg/mL	200–900 pg/mL
**Folic acid**	5.6 ng/mL	2.7–17 ng/mL
**Haptoglobin**	200 mg/dL	30–200 mg/dL
**LDH**	180 U/L	125–220 U/L
**Bilirubin (total)**	0.6 mg/dL	0.3–1.2 mg/dL
**CRP**	4 mg/L	<5 mg/L
**Erythropoietin**	253 U/L	5–25 U/L
**Virology (Parvovirus B19, HIV, HBV, HCV, Influenza A/B, CMV, EBV, SARS-CoV-2)**	Negative	—

## 3. Discussion

We describe a rare case of transient erythroid maturation arrest following heterologous COVID-19 vaccination. The temporal association with vaccination, absence of alternative etiologies, and characteristic bone marrow morphology suggest a vaccine-related, immune-mediated suppression of erythropoiesis. The first vaccine was administered on 17 April 2021, with the second on 9 June 2021; shortly afterwards the anemia occurred.

Cases of autoimmune hemolytic anemia and aplastic anemia following COVID-19 vaccination have been reported, predominantly after mRNA-based vaccines [2,3]. In contrast, PRCA-like syndromes remain exceedingly rare. Importantly, Parvovirus B19 infection—the most common cause of acquired PRCA—was carefully excluded by both serology and PCR in our patient [5,6].

Although COVID-19 infection itself has been shown to affect erythropoiesis and red blood cell morphology [7,8,9,10,11,12,13,14], our patient had no evidence of current or prior SARS-CoV-2 infection, clearly distinguishing this case from infection-related marrow changes.

We also observed mild leukocytopenia. A large Korean population-based cohort study reported an increased incidence of hematologic abnormalities, including aplastic and nutritional anemia, following COVID-19 vaccination, predominantly associated with mRNA vaccines [9]. Additionally, transient leukocyte count alterations after vaccination have been described, although the overall incidence of clinically relevant hematologic adverse events remains very low [10].

In this case report, we identified a blockage of the erythropoiesis differentiation on proerythroblast level, which is similar to that in case of PRCA following a Parvovirus B19 infection.

Pure red cell aplasia (PRCA) is identified as a normocytic, normochromic anemia secondary to an erythropoiesis defect. Characteristic is the absence of erythroblasts in the bone marrow and reticulocytopenia in the peripheral blood [5,15]. This disorder can be secondary to a viral infection, most commonly Parvovirus B19, human immunodeficiency virus (HIV), T-cell leukemia–lymphoma virus, Epstein–Barr virus (EBV), hepatitis A, B, C, and E and cytomegalovirus [5,6]. Parvovirus B19 can directly invade the progenitor erythroid cells through interaction with a cell receptor on their surface, resulting in inhibition of erythropoiesis [5,8]. The arrest of differentiation at the pronormoblast stage is characteristic. There have not been any associations to date between SARS-CoV-2 and PRCA. Several studies have reported a reduction in hemoglobin levels in COVID-19 patients [7]. Another study illustrated how SARS-CoV-2 directly or indirectly affects erythropoiesis through direct infection of erythroid precursors/progenitors with SARS-CoV-2. A subpopulation of abundant erythroid cells, CD45+ CD71+ cells, co-express ACE2, TMPRSS2, CD147, and CD26, and these can be infected with SARS-CoV-2, which can consequently lead to stress erythropoiesis [8]. In this context a link between a SARS-CoV infection and a PRCA could theoretically be supported.

On our case, a T-cell-mediated attack against hematologic precursors may be possible.

Recent research suggests that mRNA can trigger a significant activation of immune cells, leading to the release of cytokines and chemokines by upregulating toll-like receptors.

The exact pathogenetic mechanism remains speculative. Experimental studies indicate that mRNA–lipid nanoparticle vaccine platforms can induce strong innate immune activation and cytokine release, which may theoretically interfere with erythroid differentiation in susceptible individuals. In particular, lipid nanoparticles (LNPs), which are used to deliver mRNA, have also been implicated in this process. Specifically, the mRNA-LNPs employed in preclinical vaccine trials have been shown to provoke strong inflammatory reactions. The lipid nanoparticle component of the nucleoside-modified mRNA-LNP platform used by Pfizer/BioNTech and Moderna in their COVID-19 vaccines has been linked to inducing an inflammatory response in mice, resulting in the production of IL-1β and IL-6 [16,17].

Immune-mediated mechanisms, including T-cell-driven suppression of hematopoietic precursors, have been implicated in acquired cytopenias and may represent a potential, though unproven, mechanism in this case [15,17].

The favorable response to erythropoietin supports a functional rather than structural marrow defect and suggests that supportive therapy may be effective in similar cases.

**Table 3:** Molecular analysis.

Molecular Analysis	
ASXL1	wild-type
CBL	wild-type
DNMT3A	wild-type
EZH2	wild-type
JAK2	wild-type
RUNX1	wild-type
SF3B1	wild-type
SRSF2	wild-type
TET2	wild-type
TP53	wild-type
U2AF1	wild-type
ZRSR2	wild-type

The molecular analysis resulted in all genes being unmutated.

The bone marrow smear revealed an erythropoietic maturation arrest at proerythroblast level, while some of these cells were also vacuolated, similar to the findings in case of a Parvovirus B19 infection.

**Figure 2:**
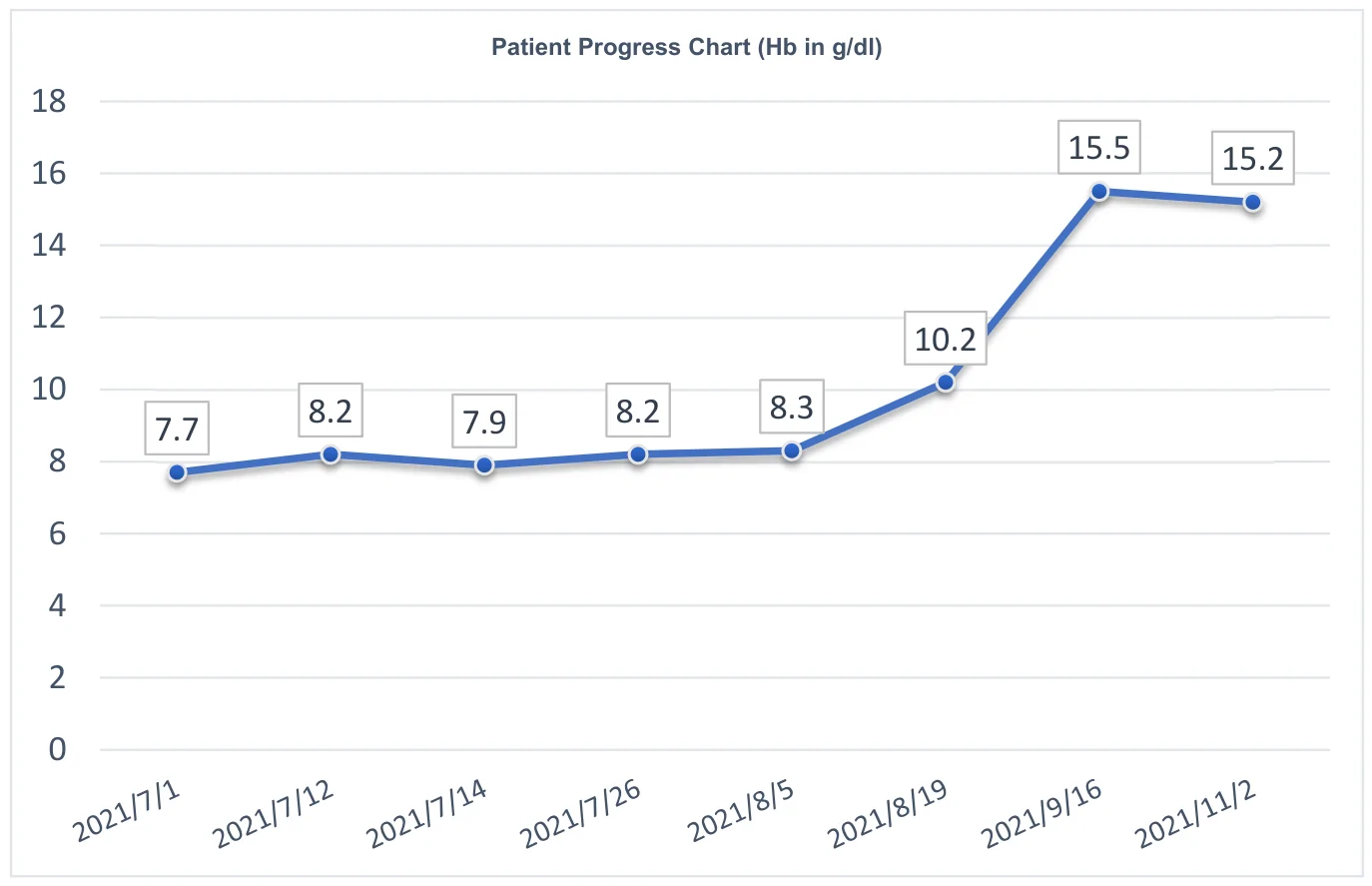
Patient progress/follow up.

At a follow up two months later, hemoglobin levels increased to 15.5 g/dL, while the white blood cells had completely regenerated (Figure 2). The normalization remains sustained after withdrawal of treatment.

## 4. Conclusions

This case illustrates a transient inhibition of erythropoiesis at the proerythroblast stage following heterologous COVID-19 vaccination, resembling a PRCA-like syndrome. Although causality cannot be definitively established, the temporal association and exclusion of alternative diagnoses support a potential vaccine-related effect.

Such events appear to be extremely rare and should not detract from the overwhelming benefits of COVID-19 vaccination.

Nevertheless, awareness of possible hematologic complications is important to facilitate timely diagnosis and management. Erythropoietin therapy may represent an effective treatment option in selected cases.

## Data Availability

The original data presented in this study are available on reasonable request from the corresponding author. The data are not publicly available due to privacy concerns.
